# Dynamic Monitoring Method of Polymer Injection Molding Product Quality Based on Operating Condition Drift Detection and Incremental Learning

**DOI:** 10.3390/polym17223025

**Published:** 2025-11-14

**Authors:** Guancheng Shen, Sihong Li, Yun Zhang, Huamin Zhou, Maoyuan Li

**Affiliations:** 1 Xi’an Modern Chemistry Research Institute, Xi’an 710065, China; 2 State Key Laboratory of Materials Processing and Die & Mould Technology, School of Materials Science and Engineering, Huazhong University of Science and Technology, Wuhan 430074, China; 3 School of Aerospace Engineering, Xiamen University, Xiamen 361005, China

**Keywords:** injection molding, product quality, drift detection, incremental learning

## Abstract

Prediction models for polymer injection molding quality often degrade due to shifts in operating conditions caused by variations in melting temperature, cooling efficiency, or machine conditions. To address this challenge, this study proposes a drift-aware dynamic quality-monitoring framework that integrates hybrid-feature autoencoder (HFAE) drift detection, sliding-window reconstruction error analysis, and a mixed-feature artificial neural network (ANN) for online quality prediction. First, shifts in processing parameters are rigorously quantified to uncover continuous drifts in both input and conditional output distributions. A HFAE monitors reconstruction errors within a sliding window to promptly detect anomalous deviations. Once the drift index exceeds a predefined threshold, the system automatically triggers a drift-event response, including the collection and labeling of a small batch of new samples. In benchmark tests, this adaptive scheme outperforms static models, achieving a 35.4% increase in overall accuracy. After two incremental updates, the root-mean-squared error decreases by 42.3% across different production intervals. The anomaly detection rate falls from 0.86 to 0.09, effectively narrowing the distribution gap between training and testing sets. By tightly coupling drift detection with online model adaptation, the proposed method not only maintains high-fidelity quality predictions under dynamically evolving injection molding conditions but also demonstrates practical relevance for large-scale industrial production, enabling reduced rework, improved process stability, and lower sampling frequency.

## 1. Introduction

Injection molding constitutes approximately 80% of all engineering plastic component production. Characterized by short cycle times, high throughput, and extensive automation, it enables rapid production of complex, tight-tolerance products [1]. However, in large-scale industrial operations, fluctuations in ambient temperature and humidity, batch-to-batch variations in raw materials, and equipment aging induce drifts in operating conditions [2]. These drifts gradually distort the mapping between process settings and product quality, rendering static models trained on historical data obsolete and degrading both prediction accuracy and online monitoring efficacy [3]. From a data-driven perspective, such drift manifests as a smooth, incremental shift in the joint probability distribution of the process’s multivariate features. Static approaches with fixed parameters cannot accommodate these distributional changes. Even memory-enabled architectures like long short-term memory (LSTM) lack mechanisms for online weight updates after deployment [4]. Consequently, there is a pressing need for a real-time monitoring solution that integrates drift detection with adaptive model updating to maintain the reliability, stability, and industrial applicability of injection molding quality predictions [5].

Numerous strategies exist for adaptively updating prediction models, including retraining [6], active forgetting [7], transfer learning [8], and meta-learning [9]. However, all presuppose the ability to detect operating condition drift promptly and accurately. Current drift-detection approaches fall into two main categories: explicit and implicit [10]. Explicit methods, which require abundant labeled data, encompass direct performance comparison and statistical hypothesis testing. Performance comparison techniques flag drift by contrasting current and historical model metrics (e.g., error rate, accuracy) [11,12]. For example, Munirathinam et al. [13] developed a unified framework that employs dual linear regression to distinguish abrupt from gradual drift and uses an adjusted box-plot method to detect outliers in both symmetric and skewed distributions. Liu et al. [14] proposed a weighted error-output recurrent Xavier echo state network with adaptive forgetting factor (WER-XESN-AFF) model, which identifies drift through analysis of historical prediction-error sequences, while Li et al. [15] updated wind power forecasting models based on variations in the root mean square error across adjacent time windows. Hypothesis-testing methods, by contrast, construct null and alternative hypotheses to quantify distributional differences between windows; Sun et al. [16], for instance, combined Jensen–Shannon divergence with Hoeffding thresholds to achieve higher detection accuracy and diversity. However, because labeled data are scarce in industrial injection-molding processes, explicit detection techniques are difficult to apply directly in such settings.

Implicit detection methods that require no labeled data fall into two principal categories: distribution-based and statistics-based approaches. Distribution-based techniques identify drift by quantifying shifts in data distributions. For example, Jain et al. [17] employed the Kullback–Leibler divergence to measure distributional changes between sliding windows, effectively detecting network-traffic drift and significantly improving anomaly detection accuracy. Castellani et al. [18] first projected high-dimensional samples into a low-dimensional embedding space, then computed distances from new samples to each class center to capture classification-impacting drift. Liu et al. [19] compared nearest-neighbor densities between current and reference data blocks, enabling drift detection in applications such as spam filtering, weather forecasting, and electricity-price prediction. However, these methods often suffer from the “curse of dimensionality” when dealing with high-dimensional data, leading to uniform distance measures or unstable computations and reduced detection efficacy. In contrast, statistics-based methods tend to be more robust. Wahab et al. [20] applied principal-component analysis (PCA) to extract eigenvalue vectors at different time points and quantified variance changes via the angles between these vectors, achieving real-time detection and tracing of malicious encrypted traffic. Hinder et al. [21] reframed drift detection as a feature-selection task, redefining drift at the feature level and proposing a series of enhancements to boost detection performance. Although these improvements have led to progress in various fields, a comprehensive framework for operating condition drift detection and model updating that can effectively handle mixed high-dimensional time series and non-time series features in large-scale injection molding production still remains a challenge.

To address the challenge of prediction performance degradation caused by operating condition drift, this study proposes a dynamic quality monitoring method for injection-molded products that integrates drift detection with incremental learning. The method first systematically analyzes the key drift inducements that affect product quality, including the operational stability of molding equipment, variations in raw material properties, process parameter fluctuations, manual interventions, and changes in the production environment. An unsupervised drift detection method based on a hybrid feature autoencoder (HFAE) is developed to enable real-time identification of drift by tracking anomalies in the reconstruction error rate within a sliding window. At the same time, a model update mechanism centered on a paired-learner structure is established. Upon drift detection, the system triggers small-batch sampling to acquire incremental labels, allowing the prediction model to be retrained and transitioned seamlessly. Finally, a closed-loop online monitoring framework is constructed, encompassing initial model training, drift detection, sample labeling, and model updating. This framework is validated using a real-world injection molding dataset, demonstrating its effectiveness and applicability in handling high-dimensional data with mixed time series and non-time series features. The proposed method offers a robust and practical solution to predictive-model degradation under dynamic industrial conditions by combining real-time drift detection via a HFAE, incremental model updating with paired learners, and a closed-loop online monitoring framework capable of handling high-dimensional mixed-feature data, thereby ensuring sustained prediction accuracy, operational stability, and reduced production costs in large-scale injection molding.

## 2. Methodology

### 2.1. Operating Condition Drift Factors Affecting Injection Molding Quality

In the injection molding production process, numerous factors influence product quality, which can be broadly categorized into the following five aspects [22]: (1) the stability and operational status of the molding equipment; (2) variations in raw material properties; (3) the setting of process parameters; (4) manual operations; and (5) fluctuations in the production environment.

#### 2.1.1. Stability and Operating Status of the Molding Equipment

**Figure 1:**
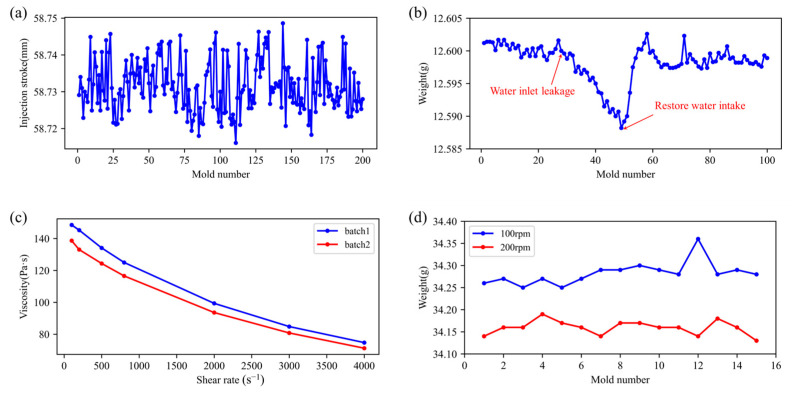
Typical operating condition drift factors affecting injection molding quality: (**a**) Changes in injection stroke during continuous production; (**b**) product weight change caused by changes in water flow rate of the mold temperature controller; (**c**) rheological curves of different batches of EP3500 materials (Mitsubishi, Japan) at different temperatures; (**d**) product weight at different screw speeds.

The stability of an injection molding system, including both the machine and its auxiliary components, directly affects product quality. Figure 1a shows that the Yizumi FF120 (Yizumi Co., Ltd., Guangdong, China) injection stroke can vary by up to 0.033 mm (0.55‰ of full stroke) during continuous runs, causing weight inconsistencies. Under sustained high temperatures and pressures, thermal and mechanical stresses accelerate wear on key parts: screw erosion leads to uneven plasticization and reduced melt flow, while check-ring wear allows melt backflow, destabilizing pressure and compromising volumetric control. Together, these degradations undermine product uniformity and dimensional accuracy.

Auxiliary equipment performance is critical to injection molding quality. In particular, the mold temperature controller regulates cavity melt flow and cooling rate, both of which directly influence surface finish and dimensional accuracy. Too-low mold temperatures impede fluidity, causing weld lines and short shots; too-high temperatures prolong cooling, slow throughput, and induce warpage or dimensional drift. In one case (Figure 1b), a water leak at the temperature-controller inlet during cycle 27 raised mold temperature and caused product weight to fall from 12.6000 g to 12.5882 g. Once the leak was fixed, weight returned to stability over about ten cycles. This incident highlights the need for real-time monitoring of auxiliary systems to maintain consistent quality and process stability.

#### 2.1.2. Variations in Raw Material Properties

Variations in plastic raw-material properties, from batch-to-batch inconsistencies, additives (e.g., recycled resin or masterbatch), or contamination, can markedly influence injection-molded part quality. Base resins from different suppliers, or even production-process changes at the same source, lead to shifts in molecular-weight distribution and melt-flow index. Because viscosity increases exponentially with molecular weight (longer chains create more entanglements and hinder flow), nominally identical materials may perform quite differently. Figure 1c presents rheological curves for several EP3500 batches measured at 260 °C on a Goettfert RG50 capillary rheometer (Goettfert, Germany). The average viscosity difference is calculated to be 6.8%, confirming noticeable discrepancies in the rheological properties of the same material across different production batches. These differences underscore the importance of material consistency and quality control in maintaining stable injection molding outcomes.

#### 2.1.3. Setting of Process Parameters

Injection molding quality depends critically on process parameters such as injection speed, hold time, screw speed, barrel temperature and back pressure. These settings, adjusted via the machine’s control panel, directly affect melt behavior and part properties. For example, when all other conditions are fixed, increasing screw speed from 100 rpm to 200 rpm reduces product weight (Figure 1d). Higher screw speeds generate more shear heating, which raises melt temperature, lowers its density and thus decreases the mass delivered per fixed injection stroke. This sensitivity underlines the importance of precise parameter control to ensure consistent outcomes.

#### 2.1.4. Manual Operation

Injection molding is a highly automated process that delivers excellent efficiency and process control, yet manual interventions, such as scheduled maintenance, unplanned repairs, mold cleaning and replacement, and anomaly handling can still exert a profound influence on product quality. For example, when production was halted for five minutes after the 10th shot and then resumed, shots 11 and 12 exhibited significantly higher part weights than those produced under steady-state conditions before gradually returning to nominal values. This weight spike arises from the material’s extended residence time in the screw barrel, which increases melt fluidity. Thus, despite its automation, injection molding remains sensitive to human-driven process disruptions.

#### 2.1.5. Fluctuation of the Production Environment

The production environment serves as a critical external factor influencing the quality of injection molding products. Ambient temperature swings (seasonal or daily) upset the thermal balance of the injection unit and mold, undermining precise temperature control and part consistency. In hygroscopic polymers like PA, elevated humidity raises moisture levels, alters melt flow and degrades mechanical properties. Likewise, mechanical vibrations or disturbances can cause fluctuations in injection pressure or screw speed, leading to quality deviations. Robust environmental monitoring and control are therefore essential to maintain stable, high-quality production.

### 2.2. Operating Condition Drift Detection Method Based on Process Data

Traditional machine learning frameworks typically comprise two primary components: a training phase where models are constructed from historical data, and a prediction phase where the trained models are applied to infer new data instances. In dynamic production settings such as injection molding, however, operating condition drift can erode prediction accuracy over time.

**Figure 2:**
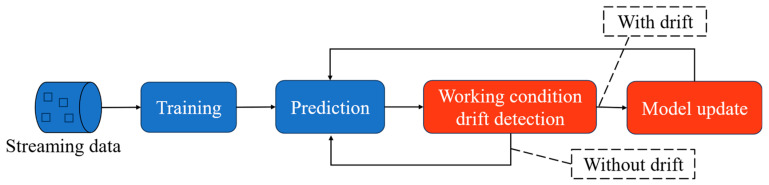
Adaptive monitoring model framework.

To overcome this limitation, we introduce an adaptive monitoring method that augments the standard framework with two additional modules: drift detection and model updating. The drift detection component continuously tracks real-time shifts in data distribution or model performance, flagging significant deviations. Once drift is identified, the model updating component adjusts the model’s structure, parameters or weights to realign it with the current data distribution and restore predictive accuracy. As shown in Figure 2, this closed-loop system, comprising training, drift detection, model updating and prediction, ensures that the model remains accurate and reliable under evolving operating conditions, making it suitable for deployment in dynamic industrial environments.

In quality prediction for injection molding products, detecting operational condition drift requires analyzing input data variations due to the scarcity of obtainable data labels. However, conventional distribution distance-based methods face challenges in handling the high-dimensional characteristics of multivariate operating condition data, often suffering from the curse of dimensionality that compromises detection accuracy. Furthermore, operational condition drift in injection molding processes typically manifests as gradual incremental changes, where subtle data variations evolve slowly over time. This characteristic makes trend analysis through extended time window observation particularly suitable. To address these challenges, this study develops an anomaly detection framework based on autoencoder (AE) architecture. The proposed methodology monitors the anomaly rate within a sliding window, triggering drift alerts when the proportion of abnormal instances surpasses a predefined threshold. As a neural network for unsupervised learning, the autoencoder comprises interconnected encoder and decoder components, demonstrating proven effectiveness in anomaly detection applications [23]. Specifically, the encoder transforms input data *x* into a compressed latent representation *z* through nonlinear dimensionality reduction, formulated as:

(1) $$z=f_{e}\left(W_{e}x+b_{e}\right)$$

where $f_{e}$ represents the mapping function of the encoder, $W_{e}$ and $b_{e}$ are the weights and bias terms of the encoder. The decoder uses this potential representation to reconstruct the original input data, expressed as:

(2) $$x\prime =f_{d}\left(W_{d}z+b_{d}\right)$$

where $f_{d}$ corresponds to the decoder’s mapping function, parameterized by $W_{d}$ and $b_{d}$. The goal of the autoencoder is to make the reconstructed data as close to the original input data as possible by minimizing the reconstruction error.

**Figure 3:**
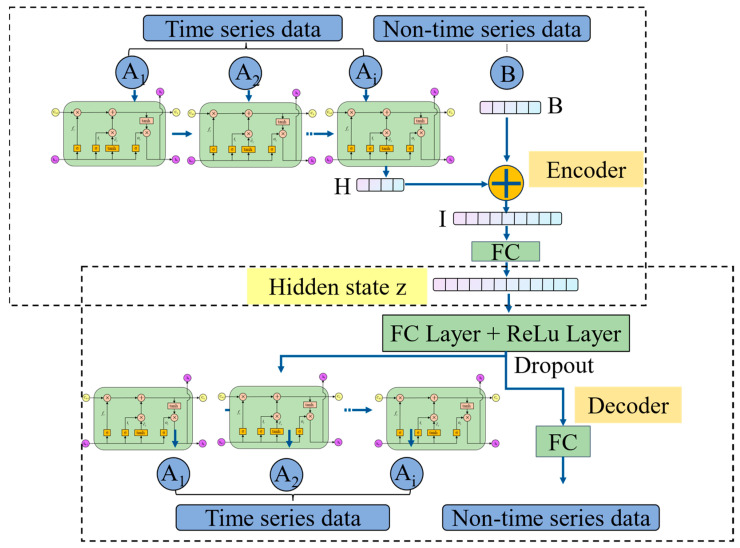
Proposed hybrid feature autoencoder.

Our previous work has shown that hybrid feature modeling enhances product-weight prediction accuracy. The previous results indicated that employing the hybrid feature modeling strategy improved the model’s prediction accuracy by 22.4%, while introducing the feature attention mechanism further increased accuracy by 11.2%. When both strategies were combined, the prediction accuracy was enhanced by 25.1%. These findings suggest that the hybrid feature modeling strategy plays a more significant role than the feature attention mechanism, and their combination yields a synergistic effect in feature representation and importance extraction. Accordingly, a hybrid feature autoencoder (HFAE) is introduced in Figure 3, deliberately omitting any feature-attention mechanism. This architectural decision reflects the differing aims of quality prediction versus reconstruction tasks; whereas feature attention in prediction models adaptively reweights inputs to minimize forecast error, the HFAE’s sole objective is to minimize its own reconstruction error. Incorporating adaptive feature weights would not improve this reconstruction criterion and thus was deemed unnecessary for the autoencoder design.

Sequential features are first encoded by a single-layer LSTM (input and output dimensions = 8), processing data in chronological order. The hidden state at the final time step is then fed into a multi-feature fusion layer, where it is concatenated with non-sequential features. This fused vector passes through a fully connected layer to yield a shared latent representation of dimension 16, capturing cross-feature correlations. In the decoder, the latent code is first restored to the fusion-layer dimension via a fully connected layer with ReLU activation and 30% dropout to introduce nonlinearity and guard against overfitting. Time series features are reconstructed through an LSTM network mirroring the encoder’s architecture, while non-time series features are recovered by a single fully connected layer. This symmetric encoder–decoder design ensures accurate reconstruction of both time-series and non-time-series inputs.

During training, the reconstruction loss is computed as the sum of the mean-squared errors for non-sequential and sequential features:

(3) $$L=L_{1}+L_{2}$$

where *L*_1_ denotes the reconstruction error for non-sequential features and *L*_2_ denotes the reconstruction error for sequential features; both are computed using mean squared error (MSE). The HFAE is trained in mini-batches of size 4 using the AdamW optimization algorithm with an initial learning rate of 0.0005 to enhance convergence stability. Once trained, the model’s reconstruction errors are used for anomaly detection; for any sample where the combined reconstruction error exceeds a predefined threshold *Q_a_*, the system will determine that the sample is abnormal. The definition of the threshold is defined as:

(4) $$Q_{\alpha }=\mu +6\sigma$$

where *μ* and σ denote the mean and standard deviation of the reconstruction errors computed over the training set, respectively.

In the method for detecting abnormal instances, the calculation of the anomaly rate relies on two types of windows. The first is a fixed window, which conceptually stores the training dataset and is used to train the HFAE model and determine the threshold *Q_a_*. The second is a sliding window, which stores real-time detection data. This window is the same size as the fixed window and moves forward with a step size of 1. The HFAE model trained on the fixed window is applied to the data within each sliding window. Abnormal instances and the corresponding anomaly rate are identified based on the threshold *Q_a_*. When the anomaly rate within the sliding window exceeds a predefined threshold, an operating condition drift is considered to have occurred.

### 2.3. Model Adaptive Update Method Based on Incremental Data

**Figure 4:**
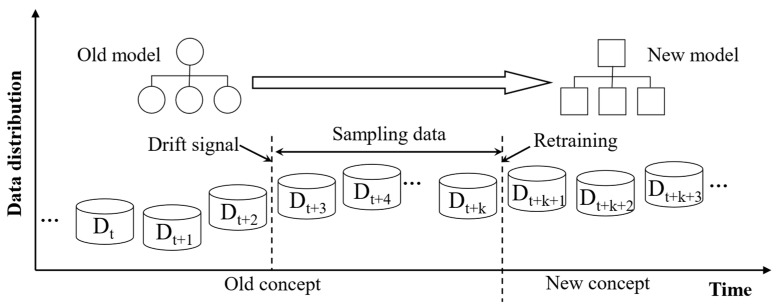
Retraining process of injection molding product weight prediction model.

When addressing operating condition drift, retraining a new model to replace the outdated one is the most direct and effective strategy. In scenarios with labeled data, the conventional time-based sampling schedule can be adapted into a drift-triggered sampling mechanism. Once a drift signal is detected, process personnel perform quality sampling on a specified batch of products, record their weight measurements, and feed these incremental data back into the model for retraining. The update workflow is illustrated in Figure 4. This approach imposes no extra burden on operators while ensuring that prediction accuracy does not degrade over time. To maintain production stability, a paired-learner scheme [24] is adopted: during new-model training the legacy model continues to handle predictions so that there is no interruption in service. After the new model is fully trained, it seamlessly replaces the old model, enabling smooth switching. Overall, this mechanism strikingly balances real-time responsiveness with sustained model accuracy, preserving consistent prediction performance without disturbing the production rhythm.

**Figure 5:**
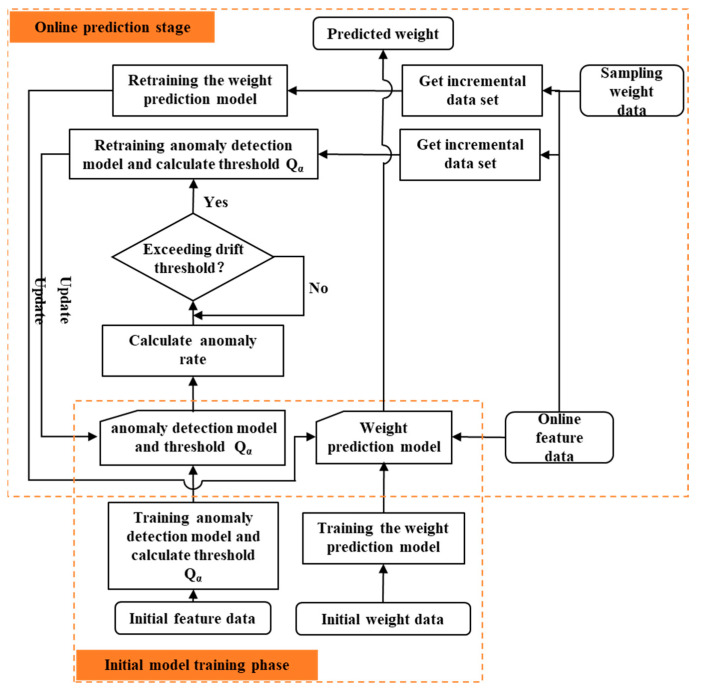
Implementation process of the model adaptive update method.

Figure 5 depicts the full adaptive-update workflow for injection-molding weight prediction, which consists of two main stages:

(1) Initial model training stage: The initial training set includes the first 100 molding cycles, selected as a practical balance between real engineering constraints and sufficient data for initial model learning. Simultaneously, an HFAE-based anomaly detector is trained and its decision threshold established to enable real-time monitoring of operating condition drift. All network architecture and hyperparameters mirror those used in the earlier work.
(2) Online prediction stage: The test data for each model begins immediately after its training range and continues until the next model update is triggered. For example, the initial model is tested from mold 100 to mold 236, and the update model-1 from mold 237 to mold 364, ensuring evaluation under true sequential production conditions. During mass production, the system continuously ingests real-time feature data. Each new data window is processed by the anomaly detector to compute reconstruction errors and an anomaly rate, which is compared against a preset threshold (0.6 in this study). At the same time, the prediction model generates weight estimates. If the anomaly rate exceeds 0.6, indicating an operating condition drift, the system alerts operators to sample 20 molds for quality inspection. After sampling, all accumulated feature data are used to retrain the anomaly detector and update its threshold, while the newly acquired feature-weight pairs are appended to the original training set to retrain the prediction model. Throughout this process, the existing models remain active, ensuring continuous drift detection and prediction until the updated models are ready for seamless deployment.

### 2.4. Experiments

This paper investigates methods for effectively detecting operating condition drift and dynamically updating the weight-prediction model in a large-scale mass-production environment, thereby ensuring the reliability of the model’s forecasts. The experimental evaluation employs the same dataset as our recent study [25] to validate the practical feasibility of the proposed approach; The detailed procedures can be found in our recent study. Table 1 summarizes the process parameters used in the experimental design.

## 3. Results and Discussions

### 3.1. Impact and Types of Operating Condition Drift Phenomenon

#### 3.1.1. Analysis of the Phenomenon of Operating Condition Drift

**Figure 6:**
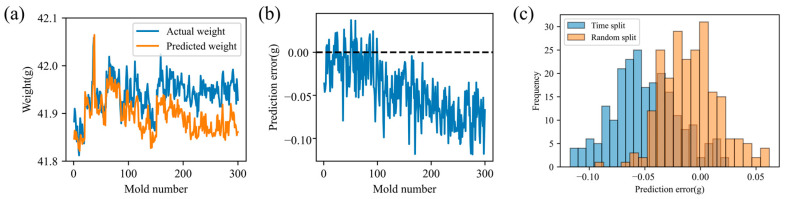
Product weight prediction results: (**a**) predicted weight and actual weight; (**b**) change in prediction error; (**c**) Comparison of prediction error distribution histograms.

To assess the impact of operating condition drift on the injection-molding quality-prediction model, we applied the previously trained model to successive production data. As shown in Figure 6a, the model delivers accurate short-term weight forecasts with minimal error; however, its performance gradually deteriorates as production continues. Figure 6b clearly demonstrates that the prediction error steadily increases over time.

Two data-partitioning strategies were implemented to assess the impact of operating condition drift: a time-series split, which preserves time series order, and a random split, which enforces distributional consistency. Because the MFA-ANN architecture depends on time series feature dependencies, a conventional feed-forward ANN was used instead to isolate the drift’s influence on predictive performance. Both strategies were trained on an identical 100-sample dataset and evaluated on the same test set. As shown in Figure 6c, the error distribution under random splitting is centered symmetrically around zero, whereas the time-series split exhibits a clear systematic bias away from zero. This bias confirms that operating condition drift markedly degrades long-term model accuracy and stability.

#### 3.1.2. Analysis of the Types of Operating Drift

Operating condition drift in the injection-molding process is characterized by shifts in the data distribution over time, which dictate whether model updating is required. Assume that the data at time point is $\left\{X,y\right\}$, where *X* idenotes the feature vector and *y* the target variable, and the operating condition at time point can be represented as the joint probability distribution $P_{t}\left(X,y\right)$. The operating condition drift between time point *t*_0_ and time point *t*_1_ is defined as:

(5) $$\exists X:P_{t_{0}}\left(X,y\right)\neq P_{t_{1}}\left(X,y\right)$$

From the perspective of probability theory, the reasons why the joint probability distribution changes over time can be divided into the following three types [5]:

(1) Input distribution drift (false drift): The marginal distribution $P\left(X\right)$ shifts while the conditional distribution $P\left(y|X\right)$ remains unchanged. The decision boundary is therefore unaffected, and only feature standardization must be reapplied.

(2) Conditional probability drift (real drift): The marginal distribution $P\left(X\right)$ stays constant, but the conditional distribution $P\left(y|X\right)$ changes. This alters the decision boundary and necessitates retraining of the model.

(3) Both $P\left(X\right)$ and $P\left(y|X\right)$ change simultaneously, combining elements of virtual and real drift. The standardization process needs to be re-performed and the model updated.

(1) **Input distribution drift analysis**

The injection-molding process exhibits pronounced time series dependencies. To intuitively compare the input-data distributions in the training and test sets, kernel density estimation (KDE) is employed to visualize their probability densities. Given a sample set $\left\{x_{1},x_{2},\ldots ,x_{n}\right\}$, the KDE of its probability density function is defined as

(6) $$\hat{f}(x)=\frac{1}{nh}\sum_{i=1}^{n}K\left(\frac{x-x_{i}}{h}\right)$$

where *n* denotes the number of samples, *h* is the bandwidth, and K(.) is the kernel function. The kernel function is used to weight the contribution of the samples. In this work, the Gaussian kernel is adopted and the bandwidth is determined using Silverman’s rule.

**Figure 7:**
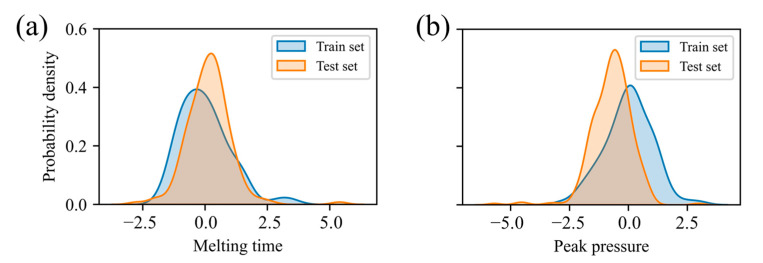
Kernel density estimation: (**a**) melting time; (**b**) return water temperature.

Figure 7 presents the KDE curves for melt time and return-water temperature after standardization, revealing pronounced distributional differences between the training and test sets. To determine whether these discrepancies are statistically significant, the Kolmogorov–Smirnov (KS) test is employed. The KS test statistic is defined as

(7) $$D_{n,m}=\sup\left|F_{1,n}\left(x\right)-F_{2,m}\left(x\right)\right|$$

where $F_{1,n}\left(x\right)$ and $F_{2,m}\left(x\right)$ are the empirical distribution functions of the training and test datasets, respectively; *n* and *m* are their sample sizes; and sup is the maximum absolute difference between these two functions. The KS test yields *p*-values of 0.017 and 1.66 × 10^−11^, both below the 0.05 significance level, confirming a statistically significant distributional shift between the training and test set, thus evidencing distribution drift in the input data.

(2) **Conditional probability drift analysis**

Detecting changes in the correlation between input features and target values is a crucial means of evaluating drift in the conditional probability distribution. When the relationship between features and target values evolves over time, it often signals an underlying shift in the conditional distribution and consequently degrades model prediction accuracy. The Pearson correlation coefficient, a classic measure of linear association between two variables, provides a static assessment of overall correlation. It is defined as:

(8) $$r_{xy}=\frac{\sum_{i=1}^{n}\left(x_{i}-\bar{x}\right)\left(y_{i}-\bar{y}\right)}{\sqrt{\sum_{i=1}^{n}{\left(x_{i}-\bar{x}\right)}^{2}}\sqrt{\sum_{i=1}^{n}{\left(y_{i}-\bar{y}\right)}^{2}}}$$

where *n* is the number of samples, $x_{i}$ and $y_{i}$ are the observed values of variables *X* and *Y*, respectively. $\bar{x}$ and $\bar{y}$ are the sample means of the corresponding variables. Since the injection molding mass production process is a time series dynamic environment scenario, the use of sliding window correlation coefficient can better capture the local change trend of correlation, thereby directly reflecting the dynamic change in conditional probability distribution $P\left(y|X\right)$ over time.

**Figure 8:**
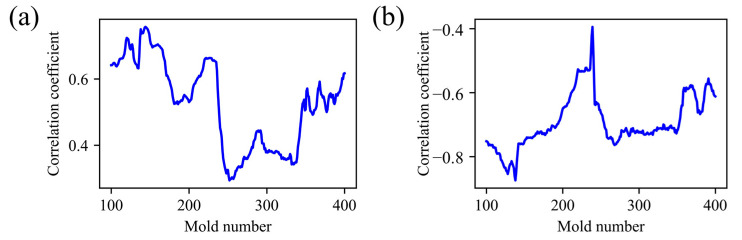
Sliding window correlation diagram: (**a**) melting time; (**b**) injection peak pressure.

Figure 8 illustrates the sliding window correlation trend between melt time and returns water temperature, using a window size equal to the initial training set (100 molds). As production progresses, the windowed correlation coefficient exhibits pronounced fluctuations. For example, between molds 237 and 251, the correlation between melt time and product weight drops sharply from 0.65 to 0.32 over only 15 consecutive measurements. This pronounced decline indicates a time series change in the feature-target relationship, underscoring the presence of conditional-distribution drift. Accordingly, the injection molding process can be classified as exhibiting joint probability distribution drift, necessitating data re-standardization and timely model updates.

### 3.2. Effectiveness of Adaptive Updating Methods for Prediction Models

**Figure 9:**
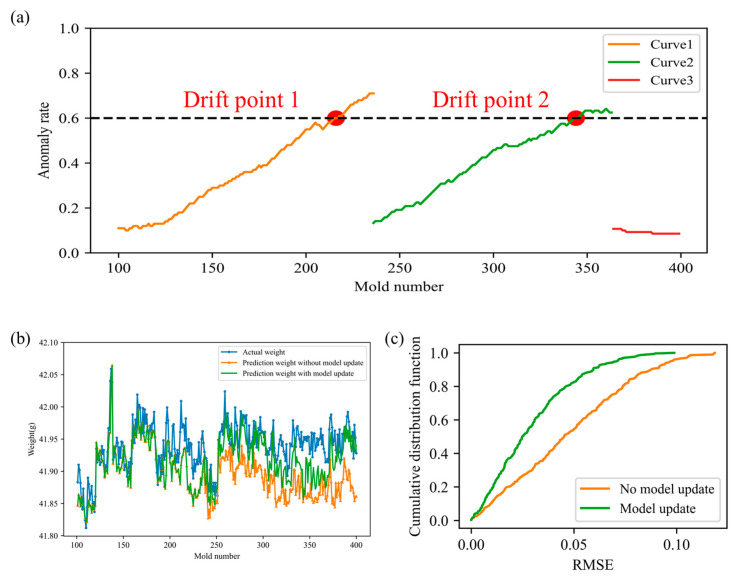
(**a**) Operating condition drift detection results; Model adaptive update results: (**b**) predicted weight and true weight; (**c**) cumulative distribution of prediction error.

Figure 9a shows the process drift detection results for the above dataset. First, the first 100 molds were used as the initial training set to train an anomaly detection model and corresponding detection thresholds. Starting from mold 101, process drift detection was performed incrementally during the forming process, and the drift detection results are shown in Curve 1. When the anomaly rate exceeded the set drift threshold (0.6), the system issued a process drift signal, marking the first drift point, which was drift point 1 (mold 216). Subsequently, the product quality of molds 217 to 236 was sampled, and the initial anomaly detection model and thresholds were used to detect and evaluate the data. After the sampling was completed, the anomaly detection model and thresholds were updated to adapt to the new data distribution, and process drift detection was performed on subsequent models. Assuming that updating the anomaly, detection model and thresholds can be completed within one model time, the updated drift detection results are shown in Curve 2. Similarly, the system detected the second drift point (drift point 2) at mold 344. Subsequently, the product quality of molds 345 to 364 was randomly inspected, and the anomaly detection model and threshold were updated again, resulting in new detection results as shown in Curve 3. During this stage, the anomaly rate of the collected data no longer exceeded the set drift threshold, and the system did not generate any new drift points.

Upon receiving the operating condition drift signal, the product weight prediction model is immediately updated, assuming that the update can be completed within one mold time. Figure 9b,c presents the product weight prediction results and their cumulative error distribution under conditions with and without model updates. After the model update, the predicted weight aligns more closely with the actual product weight, and its cumulative distribution function (CDF) curve generally lies to the left of the CDF curve without drift adaptation, indicating improved prediction performance. The prediction errors under the two scenarios were calculated separately. The RMSE with model updating is 0.0359, whereas it is 0.0556 without model updating, representing a 35.43% improvement in prediction accuracy. A paired *t*-test was conducted to evaluate whether the difference in prediction performance between the two modes is statistically significant. The resulting *p*-value is well below the significance level of 0.05, confirming a significant improvement in model performance with updating. These results demonstrate that the adaptive monitoring method for injection molding quality proposed in this study can effectively mitigate the degradation of product weight prediction performance over time.

### 3.3. Adaptive Dynamic Updating Process of Prediction Model

**Figure 10:**
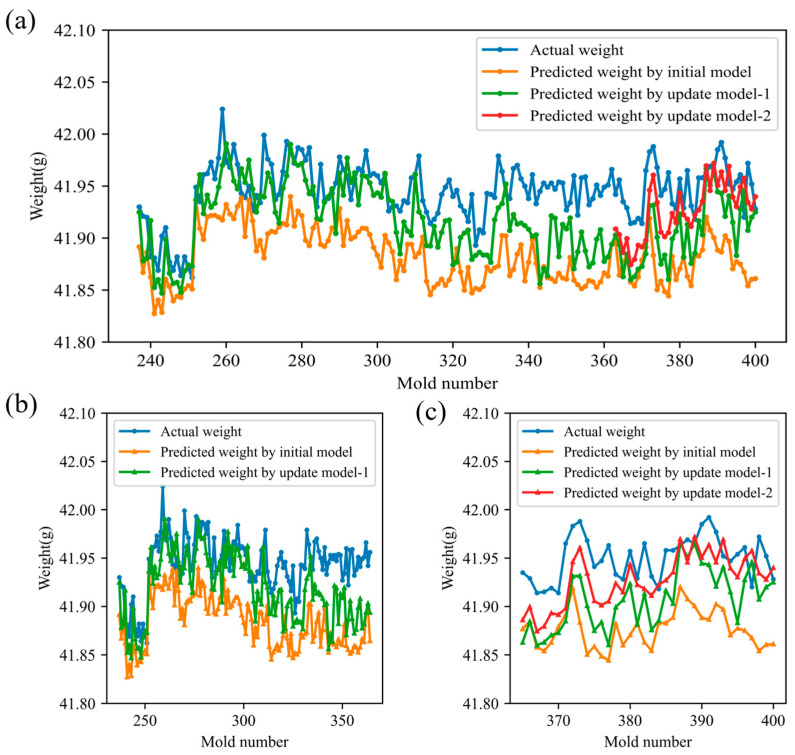
Comparison of model prediction results (**a**) All stages; (**b**) Interval 2; (**c**) Interval 3.

To evaluate the proposed adaptive quality-monitoring method’s ability to counteract performance degradation from operating condition drift, three product-weight prediction models were tracked: the initial model, update model-1 (retrained after the first drift detection), and update model-2 (retrained after the second detection). Figure 10 compares their predictions on subsequent production runs. Overall, update model-1 yields predictions closer to the true product weights than the initial model after Drift Point 1, demonstrating that model retraining effectively enhances accuracy. Following drift point 2, update model-2 further improves upon update model-1’s performance, confirming that iterative updates continue to refine predictive precision. These results indicate that the proposed method can sustain high model performance by sequentially retraining whenever operating condition drift is detected.

**Figure 11:**
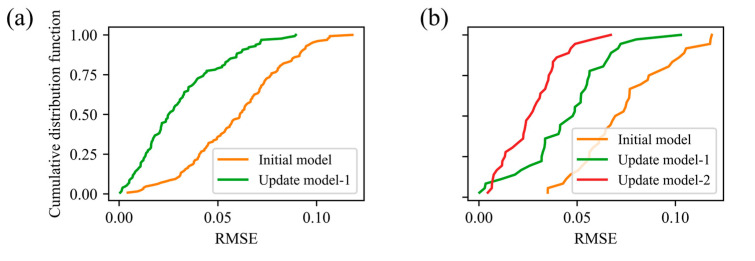
Cumulative distribution of prediction errors in different intervals: (**a**) interval 1; (**b**) interval 2.

The test set was partitioned into three intervals based on the two model-retraining events: Interval 1 (molds 101–236), Interval 2 (molds 237–364), and Interval 3 (molds 365–400). Table 2 summarizes the RMSEs for the initial model, update model-1, and update model-2 across these intervals. Examining the initial model’s performance over time reveals clear degradation: its RMSE increases from 0.0365 in Interval 1 to 0.0650 in Interval 2 and 0.0771 in Interval 3. In Interval 2, retraining yields update model-1 with an RMSE of 0.0375, and a 42.3% reduction relative to the initial model. In Interval 3, update model-1 achieves an RMSE of 0.0500 (35.2% lower than the initial model), and update model-2 further reduces the RMSE to 0.0309, representing a 60.0% improvement over the initial model. Figure 11 shows the cumulative distribution of prediction errors for each interval: after each update, the error distribution shifts markedly to the left, confirming that the adaptive retraining strategy effectively mitigates model degradation and sustains high predictive accuracy.

To further validate the proposed adaptive quality-monitoring method’s effectiveness in mitigating model-prediction degradation, independent-sample *t*-tests were conducted on prediction results from three stages: (1) the initial model’s predictions in Interval 1; (2) Update model-1’s predictions in Interval 2; (3) Update model-2’s predictions in Interval 3. Because each set of predictions is generated independently by a different model and the observations across intervals are unpaired, the independent-sample *t*-test is appropriate for assessing whether differences in predictive performance between stages are statistically significant. The test statistic is defined as:

(9) $$t=\frac{{\bar{x}}_{1}-{\bar{x}}_{2}}{\sqrt{\frac{s_{1}^{2}}{n_{1}}+\frac{s_{2}^{2}}{n_{2}}}}$$

where ${\bar{x}}_{1}$ and ${\bar{x}}_{2}$ are the sample means of the two groups of data, $s_{1}^{2}$ and $s_{2}^{2}$ are the sample variances of the two groups of data, *n*_1_ and *n*_2_ are the sample sizes of the two groups of data. The calculated *p*-values are 0.51, 0.29, and 0.15, respectively. Before performing the *t*-test, the Levene test confirmed homogeneity of variance. Together, these results indicate no statistically significant differences in predictive performance across the three intervals, confirming that the model’s accuracy has not deteriorated.

**Figure 12:**
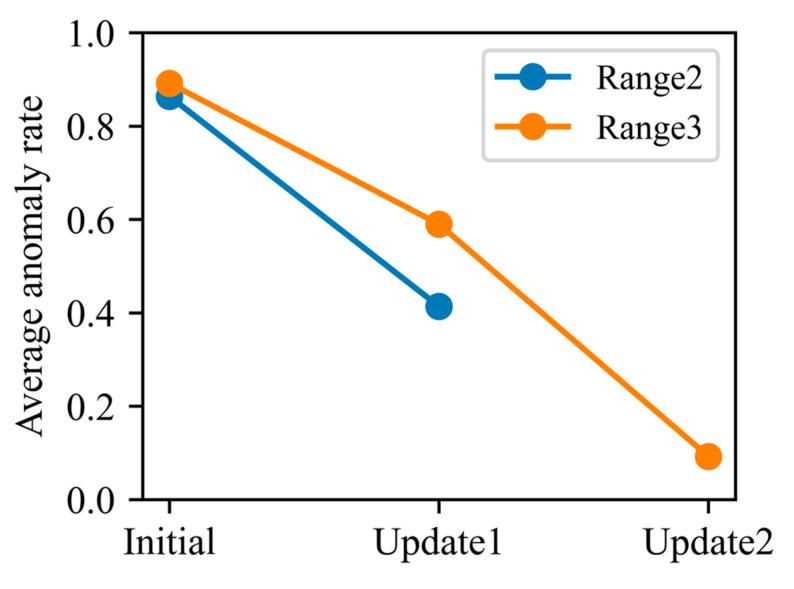
Comparison of average abnormality rates before and after model update.

From the standpoint of anomaly rates, the same test-set intervals were used to quantify how the update process narrows the distributional gap between training and test data. As shown in Figure 12, in Interval 2 the initial model’s average anomaly rate was 0.86, whereas update model-1 reduced it to 0.41. In Interval 3, the initial model’s anomaly rate stood at 0.89; update model-1 lowered this to 0.59, and further retraining to update model-2 drove the rate down to just 0.09. These findings demonstrate that each model update substantially diminishes the divergence between training and test distributions and effectively mitigates performance degradation caused by operating condition drift.

## 4. Conclusions

To mitigate the degradation of prediction performance caused by operating condition drift in the injection-molding process, this paper introduces a dynamic quality-monitoring framework that integrates real-time drift detection with incremental learning. An anomaly detection rate computed over a sliding window identifies shifts in the process distribution as they occur. When a distribution change is detected, the product-quality prediction model is incrementally updated to counteract performance loss over time. Unlike conventional static modeling strategies that assume fixed data distributions, the proposed framework continuously adapts to new operating conditions, enabling long-term stable prediction performance in real manufacturing environments.

Compared with a static baseline model, our method achieves a 35.43% improvement in overall prediction accuracy. In particular, following the first update, the model’s RMSE decreases from 0.0650 to 0.0375 (a 42.3% gain in accuracy), and the anomaly rate falls from 0.86 to 0.41. Following the second update, RMSE progressively decreases from 0.0771 to 0.0500 and then to 0.0309, corresponding to a cumulative accuracy improvement of 60.0%. Meanwhile, the anomaly rate falls from 0.89 to 0.59 and ultimately to 0.09. These results show that successive incremental updates effectively realign the training and testing distributions, substantially improving the model’s resilience to long-term drift and ensuring stable predictive capability across multiple production cycles. Overall, the proposed framework overcomes the rapid performance decay observed in static models, demonstrates strong applicability to industrial large-batch manufacturing scenarios, and provides a practical, adaptive solution for online quality monitoring, early fault warning, and process optimization under dynamic operating conditions.

## Figures and Tables

**Table 1:** Experimental process parameters [25].

Process Parameters	Unit	Values
Injection pressure	MPa	150; 150; 150
Injection speed	mm/s	150; 130; 110
Injection position	mm	70; 35
VP switch position	mm	15
Holding pressure	MPa	30
Holding time	s	0.5
Cooling time	s	25
Barrel temperature	°C	200; 210; 205; 175
Mold temperature controller temperature	°C	45

**Table 2:** RMSE values before and after weight prediction model update.

	Interval 1	Interval 2	Interval 3
Initial model	0.0356	0.0650	0.0771
Update model-1	×	0.0375	0.0500
Update model-2	×	×	0.0309

## Data Availability

The raw data supporting the conclusions of this article will be made available by the authors on request.
